# A161 IMPACT OF EARLY-ONSET COLORECTAL CANCER ON DISABILITY-ADJUSTED LIFE YEARS IN CANADA: 1990 TO 2019

**DOI:** 10.1093/jcag/gwad061.161

**Published:** 2024-02-14

**Authors:** I Stukalin, M Gupta, K Buhler, C Ma

**Affiliations:** University of Calgary, Calgary, AB, Canada; University of Calgary, Calgary, AB, Canada; University of Calgary, Calgary, AB, Canada; University of Calgary, Calgary, AB, Canada

## Abstract

**Background:**

Colorectal cancer is the third most common malignancy and remains a leading cause of potentially preventable morbidity and mortality. Early-onset colorectal cancer (EOCRC) is a growing public health focus, particularly in North America, where incidence rates have increased over time.

**Aims:**

Recognizing that EOCRC affects patients in the prime of their life, we aimed to estimate the impact of EO-CRC on disability-adjusted life years (DALYs) lost in Canada between 1990 and 2019.

**Methods:**

We used the Global Burden of Diseases (GBD) Study to assess temporal trends in incidence, mortality and DALYs for EOCRC (patients ampersand:003C50 years old) in Canada between 1990 and 2019. Point estimates are available from http://ghdx.healthdata.

org/gbd-results-tool. Rates were estimated per 100 0000 individuals at risk and stratified by age and sex. Annual percentage changes (APC) were estimated using joinpoint regression with 95% confidence intervals (CIs).

**Results:**

In 2019, the incidence, mortality and corresponding DALYs rates for EOCRC were 15.67 (95% CI 11.58, 20.81), 3.19 (95% CI 2.81, 3.61), and 161.88 (95% CI 142.50, 183.53) per 100,000 individuals, respectively. Overall incidence increased significantly during the study period by 1.12%/year (95% CI 0.91%, 1.62%). An analysis of the temporal trends demonstrates that the most significant increase in incidence in EOCRC occurred between 2000 - 2006 with an APC of 3.19% (95% CI 2.40%, 3.99%), which was primarily driven by an increased incidence in men. Mortality (APC 3.30%, 95% CI 2.41%, 4.19%) and DALYs (APC 3.28%, 95% CI 2.34%, 4.22%) for EOCRC also significantly increased for males between 2001 - 2006.

**Conclusions:**

Our study reveals a substantial burden in early-onset colorectal cancer in Canada, with a significant increase in incidence over time and over 160 DALYs/100,000 population.

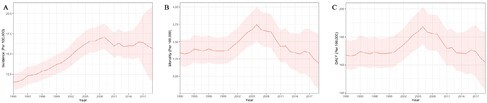

**Figure 1:** Incidence (A), Mortality (B) and DALYs (C) for EOCRC in Canada between 1990 and 2019 with 95% CIs.

**Funding Agencies:**

None

